# Generation of GCaMP6s-Expressing Zebrafish to Monitor Spatiotemporal Dynamics of Calcium Signaling Elicited by Heat Stress

**DOI:** 10.3390/ijms22115551

**Published:** 2021-05-24

**Authors:** Fengyang Li, Yong Long, Juhong Xie, Jing Ren, Tong Zhou, Guili Song, Qing Li, Zongbin Cui

**Affiliations:** 1 College of Fisheries and Life Science, Dalian Ocean University, Dalian 116023, China; LiFengyang163@163.com; 2 State Key Laboratory of Freshwater Ecology and Biotechnology, Institute of Hydrobiology, Chinese Academy of Sciences, Wuhan 430072, China; xiejuhong@inb.ac.cn (J.X.); Tongzhou_2020@163.com (T.Z.); guilisong@ihb.ac.cn (G.S.); qli@ihb.ac.cn (Q.L.); 3 College of Advanced Agricultural Sciences, University of Chinese Academy of Sciences, Beijing 100049, China; 4 Guangdong Provincial Key Laboratory of Microbial Culture Collection and Application, State Key Laboratory of Applied Microbiology Southern China, Institute of Microbiology, Guangdong Academy of Sciences, Guangzhou 510070, China; renjing@ihb.ac.cn; 5 The Innovative Academy of Seed Design, Chinese Academy of Sciences, Beijing 100101, China

**Keywords:** calcium signaling, GCaMP6s, transgenic zebrafish, heat stress, heat shock response

## Abstract

The ability of organisms to quickly sense and transduce signals of environmental stresses is critical for their survival. Ca^2+^ is a versatile intracellular messenger involved in sensing a wide variety of stresses and regulating the subsequent cellular responses. So far, our understanding for calcium signaling was mostly obtained from ex vivo tissues and cultured cell lines, and the in vivo spatiotemporal dynamics of stress-triggered calcium signaling in a vertebrate remains to be characterized. Here, we describe the generation and characterization of a transgenic zebrafish line with ubiquitous expression of GCaMP6s, a genetically encoded calcium indicator (GECI). We developed a method to investigate the spatiotemporal patterns of Ca^2+^ events induced by heat stress. Exposure to heat stress elicited immediate and transient calcium signaling in developing zebrafish. Cells extensively distributed in the integument of the head and body trunk were the first batch of responders and different cell populations demonstrated distinct response patterns upon heat stress. Activity of the heat stress-induced calcium signaling peaked at 30 s and swiftly decreased to near the basal level at 120 s after the beginning of exposure. Inhibition of the heat-induced calcium signaling by LaCl_3_ and capsazepine and treatment with the inhibitors for CaMKII (Ca²^2^/calmodulin-dependent protein kinase II) and HSF1 (Heat shock factor 1) all significantly depressed the enhanced heat shock response (HSR). Together, we delineated the spatiotemporal dynamics of heat-induced calcium signaling and confirmed functions of the Ca^2+^-CaMKII-HSF1 pathway in regulating the HSR in zebrafish.

## 1. Introduction

Temperature is the master ecological factor that pervasively affects all cellular processes [1]. For all living organisms, the maintenance of a narrow thermal window is evolutionarily favored to minimize the energy costs associated with plasticity and small changes in temperature represent a challenging problem for survival [2,3]. In the context of global climate change, fishes are prone to be affected by rapid changes in environment temperatures [2,4]. Thermal stress can directly disrupt the higher order structures and hence the functions of macromolecules including proteins, nucleic acids and lipoprotein membranes [2,5]. Furthermore, metabolic perturbations elicited by thermal stress can generate reactive oxygen species (ROS), which can cause oxidative damages to biomolecules and impair the physiological functions of cells [2,5]. Accumulation of the molecular and cellular damages may decrease physiological performance and ultimately result in mortality of the organism [6]. In response to heat stress, cells can initiate a heat shock response (HSR) to minimize the adverse effects and restore cellular homeostasis [3]. HSR is conserved across kingdoms, characterized by rapid and robust induction of the heat shock proteins (HSPs) [2]. HSPs play critical functions in the defense of protein homeostasis, the refolding of denatured proteins, and the breakdown and replacement of the proteins that are unrepairable [3,5,7].

Against the background of global warming, investigating the mechanisms underlying HSR regulation is of great interest. HSR is well known to be regulated at the level of transcription by the heat shock transcription factors (HSFs) and HSF1 is the master regulator of the HSR in eukaryotes [8,9,10]. HSF1 is constitutively expressed as an inactive monomer under normal temperature; upon heat stress, it trimerizes and binds to the heat shock element (HSE) in the promoter of the HSP genes [11]. Promoter-bound HSF1 recruits general transcription factors and co-factors to dramatically increase the transcription of the HSP genes [12,13]. The transcriptional activities of HSF1 are tightly regulated by mechanisms including trimerization, post translational modifications (PTMs) like acetylation, phosphorylation and sumoylation, and feedback from HSPs [13,14,15]. For example, phosphorylation at serine 230 by CaMKII was reported to promote the transcriptional activity of HSF1 [16]. Furthermore, there are also HSF1-independent heat shock-induced genes and the serum response factor appears to transiently induce cytoskeletal genes upon heat stress in mammalian cells [9].

Since organisms are prone to strong thermal variations, they must sense early moderate temperature increments to induce HSR in anticipation of upcoming noxious temperatures [17]. Several temperature-sensitive ion channels belonging to the transient receptor potential (TRP) superfamily called thermoTRP channels act as thermal sensors in a wide variety of animal species [18,19]. Among the thermoTRP channels, TRPV1, TRPV2, and TRPM3 can be activated by noxious heat [20]. Zebrafish Trpv1 acts as a molecular sensor of environmental heat (≥25 °C) that is distinctly lower than the sensitivity of the mammalian form (≥42 °C) but consistent with the thresholds measured in behavioral assays [21]. In an immortalized human keratinocyte cell line, Ca^2+^ influx through TRPV1 was suggested to signal the heat stress and mediate the up-regulation of a heat-shock-induced gene, MMP-1 [22]. In the post-synaptic muscle cells of *Caenorhabditis* *elegans*, the HSR was indicated to be induced by neuronal signaling through calcium-dependent activation of HSF-1 [23]. Plenty of evidence indicates that Ca^2+^-calmodulin plays critical roles in regulating HSR in plants [17,24,25,26]. These findings indicate the functions of calcium signaling in heat stress sensation and HSR regulation. Considering the roles of CaMKII in activating HSF1 [16], the HSR is probably regulated by the Ca^2+^-CaMKII-HSF1 signaling pathway.

Ca^2+^ is a highly versatile intracellular messenger that can regulate multiple cellular processes [27,28]. During calcium signaling, the low concentrations of Ca^2+^ in the cytoplasm are first increased through channel-mediated influx from external medium or release of the internal stores. The cytosolic Ca^2+^ can bind to numerous sensors and effectors to change their conformations and activities, and thus initiate the downstream signaling events [29]. Different cell type can assemble Ca^2+^-signaling systems with very different spatial and temporal dynamics through expressing a unique set of components from the extensive Ca^2+^-signaling toolkit [28]. Depending on cell type and the nature of stimulation, Ca^2+^ signals can be transient or oscillatory, and can occur as localized or global events [30]. So far, the vast majority of studies on calcium signaling have been conducted on isolated single cells and cultured cell lines [31], investigating the spatiotemporal dynamics of heat-induced calcium signaling in vivo would provide new insights into the mechanisms underlying HSR regulation. In recent years, the development of the GECIs like the green fluorescent protein/calmodulin protein sensor (GCaMP) greatly facilitated in vivo calcium signaling study [32].

Zebrafish is a valuable model for multiple disciplines due to the short reproduction cycle, transparent embryos and mature genetic tools, and has been applied to investigate calcium signaling and thermal stress response [21,33,34,35]. Transgenic zebrafish lines expressing different version of GCaMP have been generated to investigate calcium signaling underlying early embryogenesis [34], neuronal activities [36,37,38] and formation of cardiac conduction system [39]. However, the spatiotemporal dynamics of heat-elicited calcium signaling has not been characterized using GCaMP transgenic zebrafish. Most of the previous studies used tissue-specific promoters to drive expression of the GCaMP reporter, therefore the obtained fish lines are not suitable for disclosing the whole-body responses of calcium signaling triggered by environmental perturbations like heat stress.

In this study, we generated a transgenic zebrafish line with ubiquitous expression of GCaMP6s, which is driven by the common carp (*Ciprinus capio*) *beta actin* (*actb*) gene promoter [40]. This transgenic fish line has a clean genetic background, as shown by a single copy of the transgene integrated in the intergenic region. We developed a protocol to characterize in vivo calcium signaling dynamics using larvae of the transgenic zebrafish and revealed the spatiotemporal patterns of the heat-stimulated calcium signaling in zebrafish larvae. Combinatory usage of LaCl_3_ and capsazepine, inhibitors of membrane calcium channels, attenuated the altitude of the heat-elicited calcium signaling. Furthermore, the role of the Ca^2+^-CaMKII-HSF1 pathway in regulating the HSR of zebrafish was confirmed by specific inhibitors and real-time quantitative PCR (qPCR) assays.

## 2. Results

### 2.1. Generation and Characterization of GCaMP6s-Expressing Zebrafish

**Figure 1:**
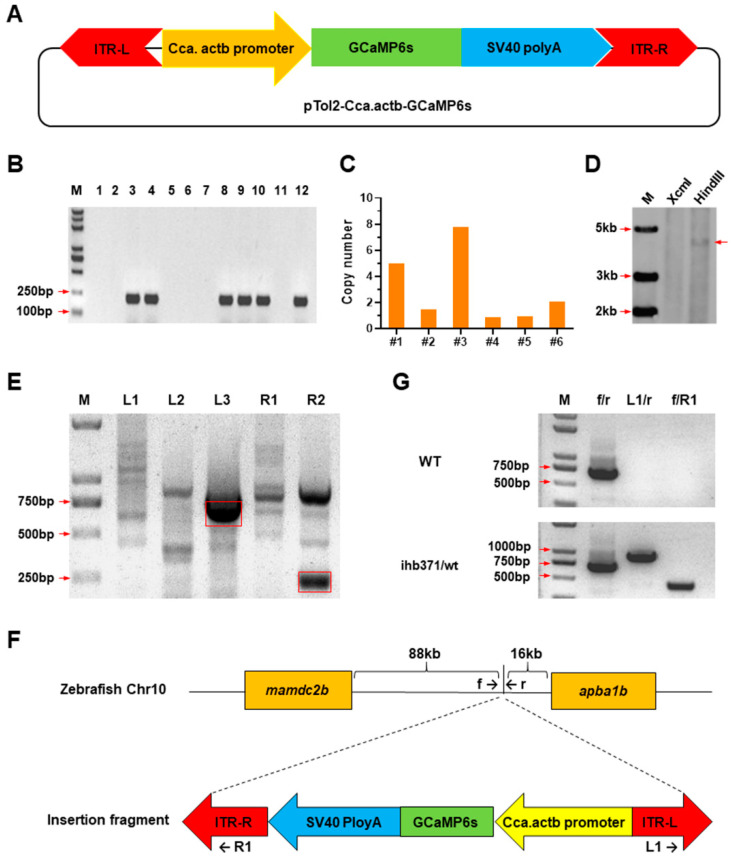
Generation of Tg[Cca.actb:GCaMP6s](ihb371Tg) transgenic zebrafish. (**A**) Schematic of the pTol2-Cca.actb-GCaMP6s construct. ITR-L and ITR-R, left and right inverted terminal repeats of Tol2 transposon; Cca.actb promoter, promoter of the common carp *β-actin* (*actb*) gene. Directions of the arrows and arrow heads are the same as the transgenic elements they represented. The transgenic elements were shown in different color for clear discrimination. (**B**) PCR screen of positive P0 individuals. M: DNA marker; 1 to 12: fish number. (**C**) Transgene copy number of the positive F1 individuals detected by qPCR assay. The x-axis represents fish number. (**D**) Southern blotting confirmed a single copy of the transgene in the genome of F1 transgenic zebrafish. The genomic DNA was digested by XcmI and HindIII, respectively. (**E**) Cloning flanking sequences of the transgene through genome walking. L1 to L3: products of the first to third round of PCR reactions using primers located on ITR-L; R1 and R2: products of the first and second round of PCR reactions using primers located on ITR-R. (**F**) Schematic of the integration site of the transgene in zebrafish genome. The insertion site is 88 kb downstream of the *mamdc2b* gene and 16 kb upstream of the *apba1b* gene on chromosome 10. The arrows indicate primers used for genotyping. (**G**) Genotyping of the transgenic zebrafish. PCR assays using primer pairs amplifying both ends of the transposon demonstrate the integrity of transgene cassette. This transgenic fish line was submitted to the China Zebrafish Resource Center (CZRC) under the accession number ihb371.

To generate a zebrafish line with ubiquitous expression of GCaMP6s, the promoter of common carp *actb* gene was used to drive expression of the transgene. The GCaMP6s -expressing cassette was located between the left and right arms of the Tol2 transposon (Figure 1A). The transgenic construct was co-injected with capped RNA of Tol2 transposase into single-cell stage zebrafish embryos. The injected embryos (P0 generation) with mosaic green fluorescence were picked out and raised to sexual maturation. Positive P0 males were identified through PCR screen (Figure 1B). F1 embryos were obtained by mating the positive P0 males with wild type (WT) females and the larvae with green fluorescence were raised to adults. Genomic DNA extracted from tail fin clips of the F1 individuals were used for detection of transgene copy number with qPCR. Transgene copy number in the genome of the analyzed F1 individuals ranged from 1 to 8 (Figure 1C). Single-copy transgene in the genome of fish #5 (Figure 1C) was confirmed by Southern blotting for the HindIII-digested genomic DNA (Figure 1D).

Genome walking based on thermal asymmetric interlaced PCR (TAIL–PCR) was performed from both arms of the transposon to identify the integration site of the transgene cassette in the genome of fish #5. Flanking sequences for both the left and right arms of the transposon were cloned and sequenced (Figure 1E). DNA sequence alignments revealed that the transgene was integrated into the intergenic region between the *mamdc2b* and *apba1b* genes, which is about 88 kilobase (kb) from the *mamdc2b* gene and 16 kb from the *apba1b* gene (Figure 1F). The integrity of the transgene cassette was confirmed by PCR assays using primers to amplify the flanking sequences and the transposon arms, f/R1 for the left arm and L1/r for the right arm (Figure 1F,G). Subsequently, this F1 male was used to establish the transgenic line, which was crossed with a WT female to generate the F2 generation. Homozygous individuals (F3 generation) were obtained by crossing the positive F2 males and females. This transgenic fish line was submitted to the China Zebrafish Resource Center (CZRC) under the accession number ihb371 (https://zfin.org/ZDB-ALT-191113-1) (accessed on 24 May 2021).

### 2.2. GCaMP6s Expression and Ca^2+^ Events in Embryos and Larvae of the Transgenic Zebrafish

**Figure 2:**
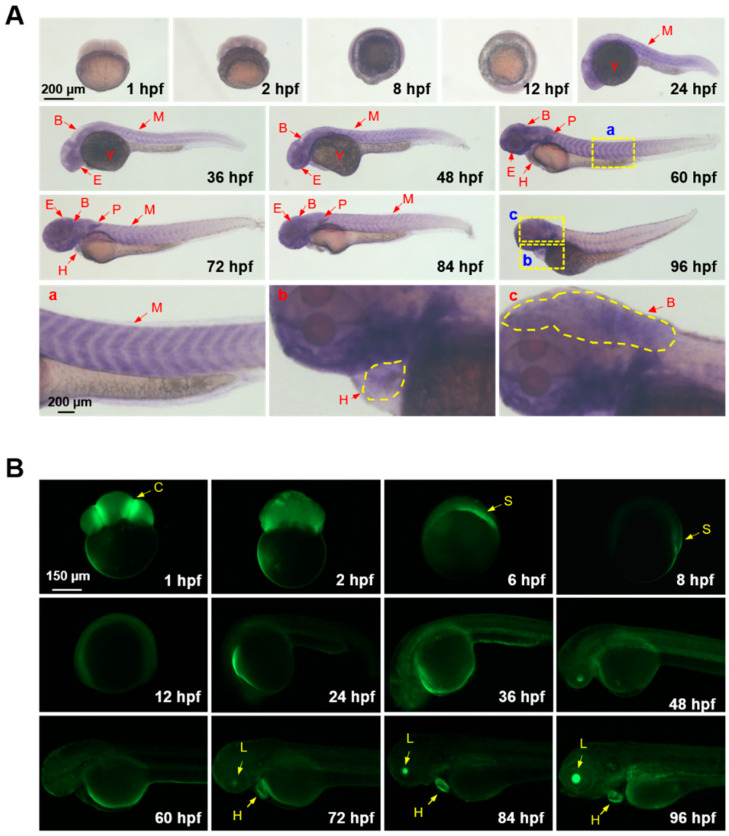
GCaMP6s expression and Ca^2+^ signaling activity in embryos and larvae of the transgenic zebrafish. (**A**) Spatiotemporal expression patterns of GCaMP6s analyzed by whole-mount in situ hybridization. The magnified images indicate the expression of GCaMP6s in the myotome (**a**), heart (**b**) and brain (**c**). (**B**) Fluorescent microscope images indicating Ca^2+^ signaling activities in the anatomical structures of zebrafish embryos and larvae. Lateral view of the early embryos (before 12 hpf) are shown. The embryos and larvae after 24 hpf were oriented as head to the left and dorsal to the top. B, brain; C, cleavage furrow; E, eyes; H, heart; L, lens; M, myotome; P, pectoral fin; S, embryonic shield; Y, yolk sac.

Transcriptional expression patterns of GCaMP6s in the embryos and larvae of the transgenic zebrafish were analyzed by whole-mount in situ hybridization (WISH). For early embryos from 1 hpf (hour post fertilization) to 8 hpf, the GCaMP6s transcripts were ubiquitously distributed, and a high level of transcription was observed in the yolk sac. From 24 to 96 hpf, GCaMP6s was widely expressed in the brain, myotomes, eyes, heart, pectoral fins and yolk sac (Figure 2A). Magnified images demonstrating the expression of GCaMP6s in the myotome, heart, and brain are also shown (a, b, and c in Figure 2A). Together, these results indicate that GCaMP6s are ubiquitously expressed, and this transgenic fish model could be used for investigating the in vivo spatiotemporal patterns of calcium signaling triggered by various environmental stressors.

The Ca^2+^ events during the embryogenesis and early larval stages of zebrafish were explored through fluorescent microscopy. Consistent with the results of a previous study [34], strong GCaMP6s fluorescence was observed in the cleavage furrows of 8-cell-stage embryos (1 hpf), indicating a high level of calcium signaling activity in this region (Figure 2B). GCaMP6s fluorescence occurred uniformly in the blastomeres of embryos at the blastula stage (2 hpf), while concentrated in the embryonic shield from 6 to 8 hpf. From 12 to 60 hpf, GCaMP6s fluorescence was relatively weak and evenly distributed. Furthermore, GCaMP6s fluorescence began to concentrate in the lens and heart of larvae at 72 hpf and the larvae at 96 hpf demonstrated very strong fluorescence intensity in these organs. The high Ca^2+^ concentrations in the lens and heart suggest that calcium signaling is important for visual perception and heart functions.

### 2.3. Establishment of a Protocol to Analyze In Vivo Activities of Calcium Signaling Triggered by Heat Stress

**Figure 3:**
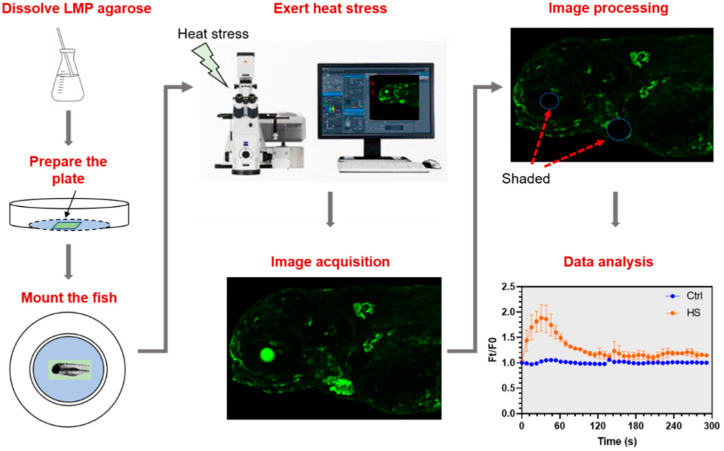
Workflow of the protocol to analyze in vivo activities of Ca^2+^ signaling triggered by heat stress. Low melting-point (LMP) agarose was melted and poured into a confocal dish. After solidification of the LMP agarose, a tiny pit was made in the middle of the agarose plane. A larva anesthetized with MS-222 was put into the pit. The dish was placed onto the stage of a confocal microscope and orientation of the larvae was adjusted. After that, Z-stack time-lapse imaging was initiated immediately upon addition of hot water into the dish. Taking into account strong basal fluorescence of the eyes and the heart, these organs were shaded before measuring fluorescence intensity. Finally, the time-lapse fluorescence intensity data were plotted to illustrate the dynamics of Ca^2+^ signaling elicited by heat stress.

A protocol was first established to reliably delineate the characteristics of calcium signaling triggered by heat stress using this GCaMP6s transgenic zebrafish line. The workflow is displayed in Figure 3. Briefly, the larvae of transgenic fish at 96 hpf were anesthetized and placed into a narrow pit made in the 1% low melting point agarose plane in a confocal dish. The larvae at 96 hpf were used for the assay due to the formation of sensory organs and cells, the pigmentation that can still be inhibited by incubation with propylthiouracil (PTU) to alleviate interference for fluorescence measurement, and the swim bladder inflation that is not completed (the larvae with inflated swim bladder are very hard to be mounted and imaged). The pit was not sealed to allow immediate and full exposure of the fish body to the exerted stressors.

The dish with the mounted fish was placed onto the stage of a confocal fluorescence microscope. The fish was moved to focus of the microscope and let to calm down from the mechanical agitation during the mounting process in 2 min and then a group of Z-stack image were taken to record the basal fluorescence intensity (F0) of the unstressed status. After that, E3 medium preheated to 40 °C was added into the dish to exert heat stress (the temperature was gradually decreased from 40 °C to 30 °C in 5 min, Supplementary Figure S1). Z-stack time-lapse imaging was initiated immediately upon adding the hot water and lasted for 5 min. The images for each time point were stacked and fluorescence intensity (Ft) was measured using the merged image. Because the eyes and heart of the transgenic fish always demonstrate strong fluorescence, they were shaded with background patches before measuring the fluorescence intensity unless otherwise indicated to avoid the disturbance to calculation of the fluorescence ratio (Ft/F0). The Ft/F0 values for the stressed and the control fish were plotted against exposure time to illustrate the effects of the stressor on calcium signaling. Except for thermal stress, this protocol can also be used to explore effects of other stressors like acidity, high salinity, and toxicants such as heavy metals by applying correspondingly formulated fish medium.

### 2.4. Spatiotemporal Dynamics of Heat Stress-Elicited Calcium Signaling in Zebrafish Larvae

**Figure 4:**
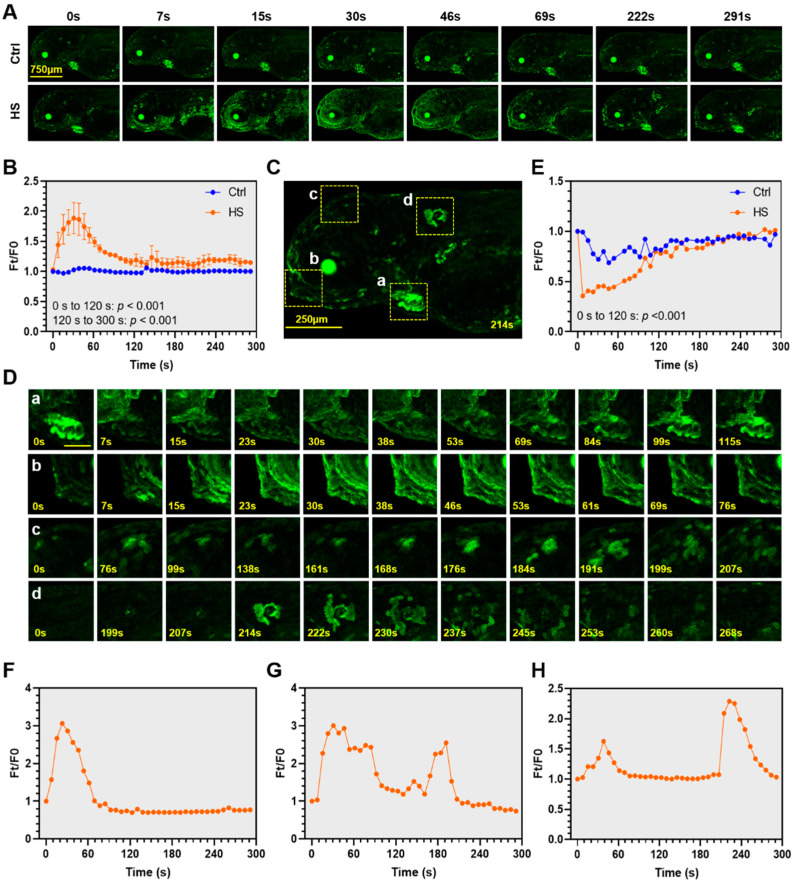
Spatiotemporal dynamics of the heat-elicited Ca^2+^ signaling in zebrafish larvae. (**A**) Representative time-lapse confocal microscope images illustrating dynamics of the heat-elicited Ca^2+^ signaling. E3 medium preconditioned at 28 °C was added to the control (Ctrl), while heat shock (HS) was exerted by adding E3 medium at 40 °C. The time points at which the images were taken are shown above the corresponding images. (**B**) Overall GCaMP6s fluorescence dynamics upon heat stress. The y-axis indicates the ratio of fluorescence intensity at different time points (Ft) to that before heat shock (F0). The x-axis indicates time duration under heat stress. Data are shown as mean ± standard error (*n* = 3). (**C**) A magnified image indicates the organs and regions (enclosed by dashed rectangles) demonstrating marked changes in GCaMP6s fluorescence upon heat stress. (**a**), heart; (**b**), region around the mouth protruding; (**c**), region above the eye; (**d**), a neuromast-like structure. (**D**) Magnified images of the organs and regions marked in (**B**). The scale bar represents 200 μm. (**E**) Exposure to heat stress transiently decreased intracellular Ca^2+^ concentration in the heart of zebrafish larvae. (**F**–**H**) Line charts demonstrating fluorescence dynamics of the representative anatomical structures shown in (**Cb**–**d**). (**F**) Region around mouth protruding (**Cb**). (**G**) Region above the eye (**Cc**). (**H**) A neuromast-like structure (**Cd**). The measurements were repeated at least three times and representative results are shown.

Spatiotemporal dynamics of calcium signaling upon heat stress were characterized using the protocol described above. Exposure to heat stress immediately triggered calcium signaling in the integument of the head, body trunk and around the eyes; while the epidermis cells of the yolk sac demonstrated no obvious response to heat stress (Figure 4A, Supplementary Movies S1 and S2). The cells demonstrating early responses to heat stress were extensively distributed, whereas those displaying late responses tended to be more concentrated (Figure 4A, from 222 s to 291 s). The heat-elicited calcium signaling was transient. The Ft/F0 value peaked at 30 s and quickly decreased to near the basal level at 120 s upon exposure (Figure 4B). After that, the Ft/F0 values of the heat-treated larvae were still significantly higher (*p* < 0.001) than those of the controls, indicating that residual calcium signaling activity could be maintained for a relatively long time (from 120 s to the end of the experiment) beyond termination of the mainstream of the heat-induced Ca^2+^ events.

To illustrate the dynamics of Ca^2+^ elevation in different anatomical structures or cell populations upon exposure to heat stress, a magnified fluorescence microscopy image of a transgenic zebrafish larva under heat stress is displayed Figure 4C. The heart (a), the region around mouth protruding (b), the region above the eye (c) and a neuromast-like structure (d) were highlighted and analyzed. It is interesting that exposure to heat stress suddenly decreased the free Ca^2+^ level in the heart, after that the GCaMP6s fluorescence intensity restored quickly to the normal level in about 100 s (Figure 4Da,E). The fluctuation of Ca^2+^ level in the heart was accompanied by the abrupt reduction and subsequent compensatory increase in heart rate (Supplementary Figure S2). These results indicate that exposure to heat stress can immediately depress heart functions through disturbing the calcium signaling activity, and that the organism can quickly restore the free Ca^2+^ level thus for the recovery from the adverse effects caused by heat shock.

The region around mouth protruding demonstrated rapid response to heat stress; a single peak of the GCaMP6s fluorescence intensity was observed at about 20 s, after that the fluorescence intensity swiftly decreased to the basal level at about 80 s upon exposure (Figure 4Db,F). The region above the eye displayed quite different response in comparison with the region around the mouth protruding, which had two fluorescence intensity peaks during the period of 5 min upon heat stress, the first one lasted from 20 sto 80 s and the second one occurred at 190 s and lasted for a shorter period (Figure 4Dc,G). The neuromast-like structure displayed in Figure 4Cd also exhibited two fluorescence intensity peaks, the first one occurred at about 40 s and the second one occurred around 220 s after the beginning of heat exposure (Figure 4Dd,H). As shown in Figure 4Dd, the second wave of calcium signaling in this anatomical structure was ignited in a single cell (199 s) located in the center and quickly spread to the surrounding cells. From 214 s to 245 s, the scope and number of the cells showing activated calcium signaling were increased while the fluorescence intensity of individual cells was decreased.

Taken together, exposure to heat stress elicited quick and transient calcium signaling in zebrafish larvae and different organs, cell populations, or anatomical structures demonstrated distinct patterns for the activity of calcium signaling.

### 2.5. Combination of Lanthanum Chloride (LaCl_3_) and Capsazepine Attenuated the Heat-Elicited Ca^2+^ Events

**Figure 5:**
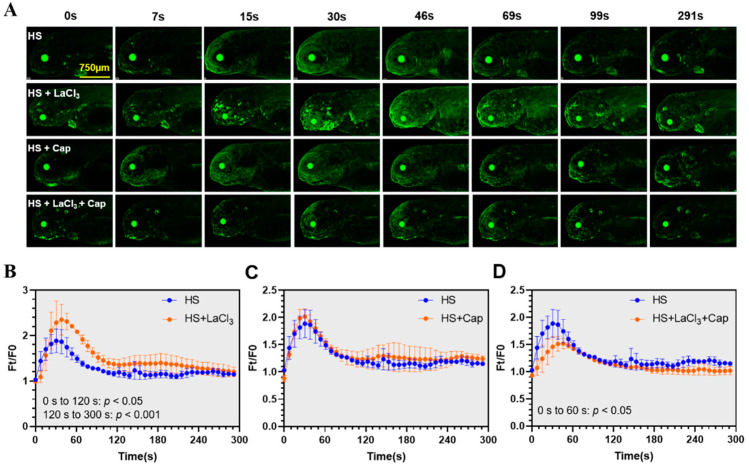
Effects of combining lanthanum chloride (LaCl_3_) and capsazepine on the heat-elicited Ca^2+^ events. (**A**) Fluorescence images of the transgenic zebrafish larvae pretreated with LaCl_3_ and capsazepine and exposed to heat stress. The larvae were pretreated with 0.2 mM LaCl_3_ (HS + LaCl_3_), 5 μM capsazepine (HS + Cap) or both (HS + LaCl_3_+ Cap). The controls were not treated with the inhibitors (HS). (**B**–**D**) Line charts indicating effects of the inhibitors on heat-induced increase of Ca^2+^ concentration. (**B**) LaCl_3_, (**C**) Capsazepine, (**D**) LaCl_3_ + capsazepine. Data are shown as mean ± standard error (*n* = 3).

After delineating the dynamics of calcium signaling upon heat stress in zebrafish larvae, the functions of calcium channels in mediating heat-triggered Ca^2+^ influx remain to be characterized. The Ca^2+^ channel blocker lanthanum chloride (LaCl_3_) was widely used to investigate the calcium signaling pathways in both plants and animals [41,42]. Capsazepine, an inhibitor of Trpv1, was also used previously to modulate the heat-induced calcium signaling [22]. We explored the effects of these two chemicals on the heat-elicited calcium signaling activities. As shown in Figure 5A,B, pretreatment with LaCl_3_ significantly enhanced the GCaMP6s fluorescence intensity upon exposure to heat stress. However, pretreatment with capsazepine displayed no discernable effects (Figure 5A,C). We then pretreated the transgenic zebrafish larvae with both LaCl_3_ and capsazepine and found that combination of these two inhibitors significantly decreased the level of GCaMP6s fluorescence intensity during the initial 60 s upon heat stress (Figure 5A,D). These results indicate that both LaCl_3_-sensitive channels and Trpv1 are involved in mediating Ca^2+^ elevation upon heat stress and simultaneous inhibition of their activities can attenuate the magnitude of the heat-elicited calcium signaling.

### 2.6. Functions of the Ca^2+^-CaMKII-HSF1 Pathway in Regulating the HSR of Zebrafish Larvae

**Figure 6:**
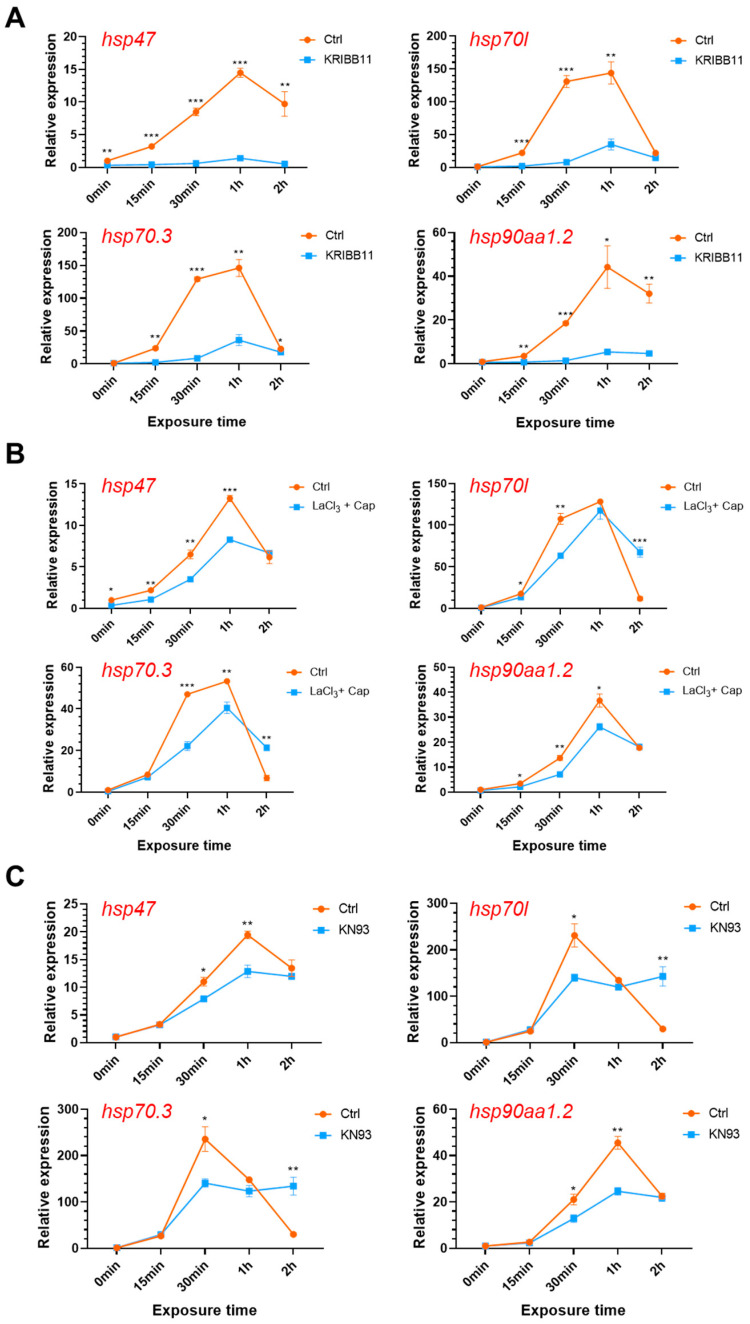
Effects of modulating the Ca^2+^-CaMKII-HSF1 pathway on expression of the *hsp* genes. Zebrafish larvae at 96 hpf were treated with the specific inhibitors for HSF1 (**A**, 10 μM KRIBB11), membrane calcium channels (**B**, 0.2 mM LaCl_3_ plus 5 μM capsazepine) and CaMKII (**C**, 10 μM KN93 phosphate) before and upon exposure to 34 °C heat stress for indicated times. Data are shown as mean ± standard error (*n* = 3). *, *p* < 0.05; **, *p* < 0.01; ***, *p* < 0.001.

Activation of HSF1 by CaMKII indicates the functions of the calcium signaling pathways in regulating the HSR [16]. Specific inhibitors for HSF1, CaMKII and membrane Ca^2+^ channels were used to characterize the contribution of the Ca^2+^-CaMKII-HSF1 pathway in regulating the HSR of zebrafish. Genes including *hsp47*, *hsp70l*, *hsp70.3*, and *hsp90aa1.2* were selected as molecular markers for the HSR. As shown in Figure 6A, expressions of the *hsp* genes in the zebrafish larvae were greatly enhanced upon exposure to 34 °C heat stress and the highest level of up-regulation was observed at 1 h upon exposure. Treatment with KEIBB11, an inhibitor of HSF1 [43] almost completely abolished the heat stress-induced up-regulation of these *hsp* genes. These results confirmed the functions of HSF1 as a master factor regulating the HSR in zebrafish. Inhibiting Ca^2+^ channels by LaCl_3_ and capsazepine (Figure 6B), and treatment with CaMKII inhibitor KN93 phosphate [44] (Figure 6C) also significantly decreased the expressions of the *hsp* genes. These results confirmed the functions of the Ca^2+^-CaMKII-HSF1 pathway in regulating the HSR of zebrafish larvae.

## 3. Discussion

The ability to quickly sense variations in environmental parameters and activate the appropriate stress responses is critical for the organisms to adapt adverse environments. How organisms sense stress signals and initiate tolerance mechanisms are fundamental biological questions. It is well known that Ca^2+^ plays pivotal roles in signaling abiotic stresses, such as osmotic stress agents, high salt, cold, heat, and oxidative stress in plants [45]. Both cytosolic and nuclear calcium-sensing pathways are involved in gene regulation and stress tolerance of plants [46]. The transient receptor potential (TRP) ion channels that mediating Ca^2+^ influx are paradigm sensors for acute noxious heat in mammals. *Trpv1*^−/−^*Trpm3*^−/−^*Trpa1*^−/−^ triple knockout mice fully lose the acute withdrawal response to noxious heat that is necessary to avoid burn injury [47]. Ca^2+^ was also reported to mediate cold sensing in insect tissues and regulate rapid cold hardening (RCH) of the ex vivo cultured midgut and salivary gland [48]. Neuronal calcium signaling in the brain of Drosophila was found to be involved in nutrient sensing and adaptation to nutrient stress [49]. Ca^2+^ is involved in osmotic stress signaling and mediates the effects of osmoregulatory hormones in fish [50]. Exposure of tilapia (*Oreochromis mossambicus*) prolactin secreting cells to hypoosmotic stress induced rise of the cytosolic Ca^2+^ concentration [51]. Furthermore, an increase in intracellular Ca^2+^ levels was found to be the primary response of many cell types to hypoxia/ischemia stress and Ca^2+^ plays essential roles in the hypoxia-induced regulation of signal transduction pathways and gene expression [52]. Calcium signaling was also suggested as a possible mechanism behind the increased locomotor response in zebrafish larvae exposed to a persistent organic pollutant [53]. However, effects of the above-mentioned stressors on calcium signaling have not been characterized using an in vivo vertebrate model. The transgenic fish line established in this study offers a valuable research model to investigate the spatiotemporal dynamics of calcium signaling elicited by various environmental stressors.

Despite considerable insights have been obtained into the functions of Ca^2+^ in sensing and signaling stresses, most of the previous studies were conducted in plants, ex vivo insect tissues and cultured mammalian cell lines. It is unclear what cells or organs of the organisms perceive the stress at first and transmit the signal to the whole body to tune stress tolerance of the organism. In this study, we generated a GCaMP6s-expressing transgenic zebrafish line and characterized the spatiotemporal dynamics of calcium signaling triggered by heat stress. Exposure to heat stress elicited quick and transient calcium signaling mainly in the integument of the head and body trunk of zebrafish larvae. The cells demonstrating the earliest responses to heat stress are extensively distributed in the epithelium of larval zebrafish, indicating that these cells are responsible for sensing the heat stress. This is consistent with the notion that environmental temperatures are detected as changes in skin temperature of the animal and free nerve endings of primary sensory neurons that lie just beneath the outer surface of the skin mainly perceive temperature changes as thermal stimuli [18].

Although the overall calcium signaling activity picked at 30 s and restored to near the basal level at about 2 min upon exposure to heat stress, different cell populations can demonstrate distinct patterns of calcium signaling upon heat stress. For example, the neuromast-like structure shown in Figure 2Cd demonstrated two peaks of calcium signaling activity. The first one occurred at 40 s and the second one occurred at 220 s after the beginning of heat exposure. The contributions of cell-specific calcium signaling for orchestration of the whole body HSR remain to be further characterized. Furthermore, the central nervous system circuits of endotherms are responsible for processing thermal afferent inputs from the skin and the body core to control the activity of thermo effectors to maintain the body temperature [54]. No significant activation of calcium signaling was observed in the central nervous system of the zebrafish larvae exposed to heat stress, despite the expression of GCaMP6s in the brain (Figure 2A). This suggests that the HSR in zebrafish larvae is independent on calcium signaling activities of the central nervous system.

In opposition to the enhancement of the overall calcium signaling activity of the whole body (exclude the eyes and heart), calcium signaling activity in the heart was inhibited and the heart rate was decreased upon exposure to heat stress. The depression in calcium signaling and heart rate lasted for 2 min, same as the duration for activation of the overall calcium signaling. After that, concentration of free Ca^2+^ in the heart restored to normal level and the heart rate was compensatively increased. The intimate relationship between the heart Ca^2+^ level and heart rate of zebrafish larvae indicates that calcium signaling plays critical roles in maintaining the contractile function of the heart. The fundamental roles of Ca^2+^ in the heart are also illustrated by the prevalence of altered Ca^2+^ homeostasis in cardiovascular diseases [55]. For humans, exercise in high ambient temperature (35 °C versus 22–25 °C) can enhance the magnitude of cardiovascular drift, a well-known phenomenon characterized by increase in heart rate and decrease in stroke volume [56]. These results indicate that heat stress can affect heart functions and thus organismal performance through modifying the calcium signaling pathways. The resemblance between the responses of zebrafish and human hearts to heat stress indicate that this transgenic zebrafish model can be used to explore the effects of genetic disorders on heart functions.

When exposed to stresses, the mechanism of calcium-induced calcium release (CICR) is responsible for the Ca^2+^ surge in the cytosol [28]. Therefore, the membrane channels medicating Ca^2+^ influx are critical for the initiation of calcium signaling upon stresses. To characterize functions of the membrane Ca^2+^ channels in regulating the heat stress-elicited calcium signaling, the transgenic zebrafish larvae were pretreated with the Ca^2+^ channel blocker LaCl_3_ and the Trpv1 inhibitor capsazepine before analyzing the GCaMP6s fluorescence. Contrary to the anticipation that both LaCl_3_ and capsazepine could decrease the multitude of the heat-induced calcium signaling, treatment with 0.2 mM LaCl_3_ enhanced Ca^2+^ influx in comparison to the controls, while incubation with 5 μM capsazepine demonstrated no effect on calcium signaling upon heat stress. Paradoxical effects of lanthanum ions on cytosolic Ca^2+^ concentration were previously reported. Lanthanum was suggested to increase the rat thymocyte cytoplasmic free Ca^2+^ concentration by enhancing calcium influx [57], and to relieve the inhibition of intracellular calcium release in the cultured chick (*Gallus gallus*) embryonic myocardial cells [58]. We further tested effect of the combination of LaCl_3_ and capsazepine and found that treatment with both inhibitors partially but significantly inhibited the heat-induced increase in Ca^2+^ concentration. These results indicate that both LaCl_3_-sensitive channels and Trpv1 are involved in gating Ca^2+^ influx upon heat stress.

The biological significance of the heat stress-induced Ca^2+^ was further explored by characterizing effects of modulating the calcium signaling cascade on the HSR. Consistent with the effects in inhibiting heat-stimulated Ca^2+^ elevation, treatment with LaCl_3_/capsazepine significantly decreased expression of the *hsp* genes upon heat stress. These results indicate that the enhancement of cytosolic free Ca^2+^ levels during the first 2 min upon exposure to heat stress has a profound effect on regulating the subsequent HSR. Treatment with KN93, an inhibitor of CaMKII also significantly reduced the heat-induced expression of the *hsp* genes. Furthermore, inhibiting the activity of HSF1 by KRIBB11 almost entirely suppressed the heat-induced expression of the *hsp* genes. Considering the functions of CaMKII in phosphorylation and activation of HSF1 [16,59], it can be concluded that the Ca^2+^-CaMKII-HSF1 pathway plays important functions in regulating the HSR and thus the ability of zebrafish to tolerate noxious heat stress. The Ca^2+^-CaMKII pathway was also reported to instantly detect decreases in temperature and trigger the downstream cold-hardening mechanisms [48]. Moreover, partial inhibition of the heat-induced expression of the *hsp* genes by LaCl_3_/capsazepine and KN93 suggests that there may be Ca^2+^-CaMKII independent pathways function to regulate HSF1 and HSR as well.

## 4. Materials and Methods

### 4.1. Chemicals

Phenylthiourea (PTU), MS-222, LaCl_3_, and DMSO were purchased from Sigma-Aldrich (St. Louis, MO, USA). Capsazepine, KRIBB11and KN-93 phosphate were obtained from Selleck (Plymouth, MI, USA). Other chemicals were obtained from Sinopharm (Shanghai, China).

### 4.2. Maintenance of Zebrafish

Wild type AB strain zebrafish were maintained in a circulating water system as previously described [60]. The water temperature was controlled at 28 °C ± 1 °C and the fish room was illuminated with 12 h/12 h light/dark cycles. The embryos were cultivated in E3 medium (5 mM NaCl, 0.17 mM KCl, 0.33 mM CaCl2, and 0.33 mM MgSO4, pH 7.2) and maintained at 28 °C using a biochemical incubator from Shanghai Jinghong (Shanghai, China). The larvae were fed with nauplii of brine shrimp and the adults were fed with frozen red worm (*Limnodrilus hoffmeisteri*) obtained from a local pet store (Wuhan, China).

### 4.3. Generation of GCaMP6s-Expressing Transgenic Construct

To generate the transgenic construct pTol2-Cca.actb-GCaMP6s (Figure 1A), Tol2-elavl3-GCaMP6s obtained from Addgene (#59531, Watertown, MA, USA) and pT2/carp_actin_P-Abcc4-Flag-carp_actin_pA constructed in our previous study [61] were double digested using *Hin*d III and *Eco*R I from Fermentas (Burlington, NJ, Canada). The desired DNA fragments were recovered through electrophoresis and subsequent gel purification using a kit from Bioer (Hangzhou, China). The common carp *β-actin* (*actb*) gene promoter was inserted into the cloning site to replace the elavl3 promoter of Tol2-elavl3-GCaMP6s. The resultant construct was confirmed by double digestion using *Eco*R I and *Xho* I (Fermentas, Burlington, NJ, Canada).

### 4.4. Preparation of Tol2 Transposase Capped mRNA and Microinjection

Capped mRNA of Tol2 transposase was prepared as previously described [62]. Briefly, pT3TS-Tol2 (Addgene, #31831, Watertown, USA) was linearized using XbaI (Fermentas, Burlington, NJ, Canada) and capped mRNA was synthesized using the mMACHINE™ T3 Transcription Kit (Thermo Scientific, Waltham, MA, USA). Fertilized eggs of zebrafish were obtained by mating the female and male adults. pTol2-Cca.actb-GCaMP6s (50 ng/µL) and capped Tol2 transposase mRNA (300 ng/µL) were co-injected into zebrafish embryos (1–2 nl/embryo) at one-cell stage using a PLI-100A microinjector from Warner Instruments (Holliston, MA, USA).

### 4.5. Transgenic Fish Screening

The injected embryos were hatched and raised to sexual maturation (the P0 generation). Genomic DNA was isolated from embryos of the P0 fish crossed with WT using a genomic DNA kit from Tiangen (Beijing, China) according to the manufacturer’s instructions. The genomic DNA was amplified through PCR using primers GCaMP6s-F/GCaMP6s-R to detect the transgene (all the primers used in this study are listed in Table S1). The positive P0 fish were individually crossed with wild type (WT) fish and the progenies were checked for GCaMP6s fluorescence using a stereo fluorescence microscope from Zeiss (Oberkochen, Germany). The F1 larvae expressing GCaMP6s fluorescence were picked out and raised to maturation.

### 4.6. Quantitative Real-Time PCR

Quantitative real-time PCR (qPCR) was performed as previously described [33] to measure copy number of the transgene integrated in the genome and to determine transcriptional expression of the heat shock protein genes.

Genomic DNA was isolated from tail fin clip of F1 fish. Concentration of the genomic DNA samples was measured using a Q5000 UV-Vis Spectrophotometer from Quawell (Sunnyvale, CA, USA). The PCR reactions were performed using 100 ng of genomic DNA in 20 μL volume. The actb1 gene was used as internal control for data normalization. Standard curves of the primers were generated using serially diluted templates to determine the amplification efficiencies (E). EGCaMP6s and Eactb1were calculated as 97.4% and 96.6%, respectively. Since the F1 fish were heterologous, copy number of the transgene was calculated as 2 * (E_GCaMP6s_^Cq_GCaMP6s_)/(E_actb1_^Cq_actb1_).

Expression of genes, including *hsp47*, *hsp70l,* and *hsp70.3*, were analyzed by qPCR to characterize heat shock response. Total RNA was extracted from the samples using TRIzol™ Reagent from Thermo Fisher Scientific (Waltham, MA, USA). First-strand cDNA was synthesized from 1 µg of total RNA using the TransScript^®^ All-in-One First-Strand cDNA Synthesis SuperMix for PCR from TransGen Biotech (Beijing, China) according to the manufacturer’s instructions. Our previous study revealed that *tpma* and *tnnt3b* were the most stable genes upon heat stress (at 34 °C) among the characterized candidates [33]. Therefore, the geometric average of their relative expressions was calculated and used as the normalization factor for calculating gene expression.

### 4.7. Southern Blotting

Southern blotting was performed according to our previous protocol [63] to confirm single copy integration of the transgene in the genome. Briefly, genomic DNA extracted from fin clip of the F1 fish that was revealed to carry single copy of transgenes was digested with XcmI and HindIII (Fermentas, Burlington, NJ, Canada) respectively at 37 °C overnight (a unique restriction site in the vector). The probe was amplified from the pTol2-Cca.actb-GCaMP6s construct using primers GCaMP6s-F/GCaMP6s-R. The probe was labeled with the DIG High Prime DNA Labeling and Detection Starter Kit II from Roche (Basel, Switzerland).

### 4.8. Genome Walking

Genome walking from both arms of the transposon was performed as previously reported [64,65,66] to clone the flanking sequences and to determine the genomic integration site of the transgene. The genomic DNA of the F1 individual that was revealed to carry a single copy of transgene by both qPCR analysis and Southern blotting was used as the template for the PCR assays. The reverse primers L1 to L3 and the forward primers R1 to R3 (Table S1) were designed according to the left and right arm sequences of the transposon. Each of the degenerate primers used in our previous study (AD5 to AD7) [66] was paired with the sequence specific primers in PCR assays to isolate the flanking sequences. The specific DNA fragments from the third or second round of PCR reactions were purified and cloned into the pMD18-T cloning vector (Takara, Kusatsu, Japan) and subjected to DNA sequencing. The resulted DNA sequences were blasted against the zebrafish genomic sequence deposited in Ensembl (http://www.ensembl.org/) (accessed on 1 March 2021) and the hit sequence with the highest score was used to identify the transgene integration site.

### 4.9. Whole Mount In Situ Hybridization

The templates for RNA probes synthesis were amplified by PCR using primers containing T3 or T7 promoter sequence, Anti_RNA-F/Anti_RNA-R for the antisense probe and Sen_RNA-F/Sen_RNA-R for the sense probe. The antisense and sense probes were transcribed from the corresponding template using T7 and T3 RNA polymerase, respectively. Digoxigenin-11-UTP from Roche (Basel, Switzerland) was incorporated in the transcription reaction to label the probes. Whole-mount in-situ hybridization assays of zebrafish embryos and larvae were performed following a previously described protocol [67].

### 4.10. Fluorescence Microscopy

To investigate the GCaMP6s fluorescence patterns of the transgenic zebrafish during early development, the embryos and larvae at different development stage were observed under a fluorescence microscope (Axio observer7) from Zeiss (Oberkochen, Germany). Phenylthiourea was added into the E3 medium to a concentration of 0.003% to prevent pigmentation. The embryos were manually dechorionated and anesthetized with 160 mg/L MS-222 before fluorescence microscopy. Z-stack imaging was performed at 7 µm intervals and a total of 20 slices were stacked for the final image.

Z-stack time-lapse imaging was performed using a Leica (Wetzlar, Germany) SP8 confocal microscope to characterize the effects of heat stress on Calcium signaling. To mount the fish for imaging, low-melting point (LMP) agarose (Sigma, St. Louis, MO, USA) was melted in E3 medium to a concentration of 1% and poured into a glass bottom confocal dish. A narrow slot was dug in the middle of the LMP agarose plate using a sharp scalpel and an anesthetized larva was placed into the slot to allow full exposure to heat stress. The dish was placed onto the stage of the confocal microscope and body of the fish was adjusted for the right orientation. After that, an image representing the basal Calcium signaling activity was taken before heat stress. To exert heat stress, the medium in the dish was sucked out using a Pasteur pipette and 1 mL E3 medium (supplied with MS-222, with or without inhibitors) preheated to 40 °C was gently added into the dish. Then, imaging was initiated immediately upon adding of the heat water. Z-stack imaging was conducted with 21 µm intervals and a total of 12 slices were acquired and stacked for the final image. Imaging of each fish was incessantly continued for 5 min and the time-lapse images were converted into a video.

### 4.11. Fluorescence Intensity Measurement and Analysis

The stacked images were subjected to GCaMP6s fluorescence intensity measurements. To characterize Calcium signaling of specific organs and regions, the regions of interest were defined and the fluorescence intensities for the time-lapse images of each fish were automatically extracted. Fluorescence intensities of the eyes and heart were subtracted from the total intensity since they constitutively demonstrated strong fluorescence. Relative fluorescence intensity was calculated by dividing the intensity at different time points under stress (Ft) by the basal intensity (F0) and plotted against the exposure time to illustrate the dynamics of Calcium signaling.

### 4.12. Heat and Chemical Treatments

To characterize roles of Calcium signaling in regulating the HSR, wild type zebrafish larvae developed to 96 hpf under 28 °C were exposed to 34 °C for different time durations in the presence of the inhibitors. For heat exposure, the larvae were randomLy assigned into 60-mm petri dishes (40 larvae per dish) 1 day before treatment. The larvae were preloaded with the inhibitors for 1 h at 28 °C before exposure to 34 °C After that, the larvae were transferred into E3 medium (containing the corresponding inhibitor: 10 μM KRIBB11, 0.2 mM LaCl_3_ and 5 μM capsazepine, 10 μM KN93 phosphate) preheated to 34 °C. For medium changing, the old medium was by filter by a small mesh and the larvae were quickly flushed down from the mesh using the new medium. The dishes were put into a water tank connected to an ARCTIC A40 Refrigerated Circulator from Thermo Fisher Scientific (Waltham, MA, USA) for temperature control. After treated for desired time durations, the larvae were collected and subjected to total RNA extraction and gene expression was determined through qPCR assays.

### 4.13. Statistical Analysis

Statistical analyses were performed using R (v4.0.3). To analyze the effects of various treatments on intensity of the calcium signaling (Ft/F0), the time course data were separated into two parts according to the transient nature of the heat-elicited calcium signaling. The first part was from 0 s to 120 s (or 60 s) and the second part was from 120 s (or 60 s) to 300 s. Significance of the difference between the control and treatment was analyzed by Kolmogorov–Smirnov test. The difference in expression of the *hsp* genes upon heat stress and inhibitor treatments was analyzed by independent-samples T Test.

## Data Availability

The data presented in this study are available in article and Supplementary Materials.

## Supplementary Materials

The following are available online at https://www.mdpi.com/article/10.3390/ijms22115551/s1, Figure S1 Temperature of the fish medium during the process of time-lapse imaging. Temperature of the medium was measured using a digital probe thermometer. Data are shown as mean ± standard deviation (n = 3). Figure S2 Heart rate of the zebrafish larvae exposed to heat stress decreased at first and then compensatively increased. Heart rates (beat/min) at the indicated time points were compared with that before exposure to heat stress (0 min). Data are shown as mean ± standard deviation (n = 3). **, *p* < 0.01. Table S1 Information of primers used in this study. Supplementary Movie S1 Movie for the time lapse photos of the control larvae (Figure 4A ctrl). Supplementary Movie S2 Movie for the time lapse photos of the heat-treated larvae (Figure 4A HS).

## Abbreviations

ROS	reactive oxygen species
HSR	heat shock response
HSP	heat shock protein
HSF	heat shock transcription factor
HSE	heat shock element
PTM	post translational modification
CaMKII	calcium/calmodulin-dependent protein kinase II
TRP	transient receptor potential
GECI	genetically encoded calcium indicator
GCaMP	green fluorescent protein/calmodulin protein
qPCR	real-time quantitative PCR
WT	wild type
TAIL–PCR	thermal asymmetric interlaced PCR
LaCl_3_	lanthanum chloride
