# Supplementary material for: Generation of GCaMP6s-Expressing Zebrafish to Monitor Spatiotemporal Dynamics of Calcium Signaling Elicited by Heat Stress
Source: Int J Mol Sci. 2021 May 24;22(11):5551. doi: 10.3390/ijms22115551 (PMC8197303; doi:10.3390/ijms22115551)
Supplement: Supplementary file 1 [file ijms-22-05551-s001.zip › Supplementary Figures S1 and S2.pdf]

Supplementary Figures

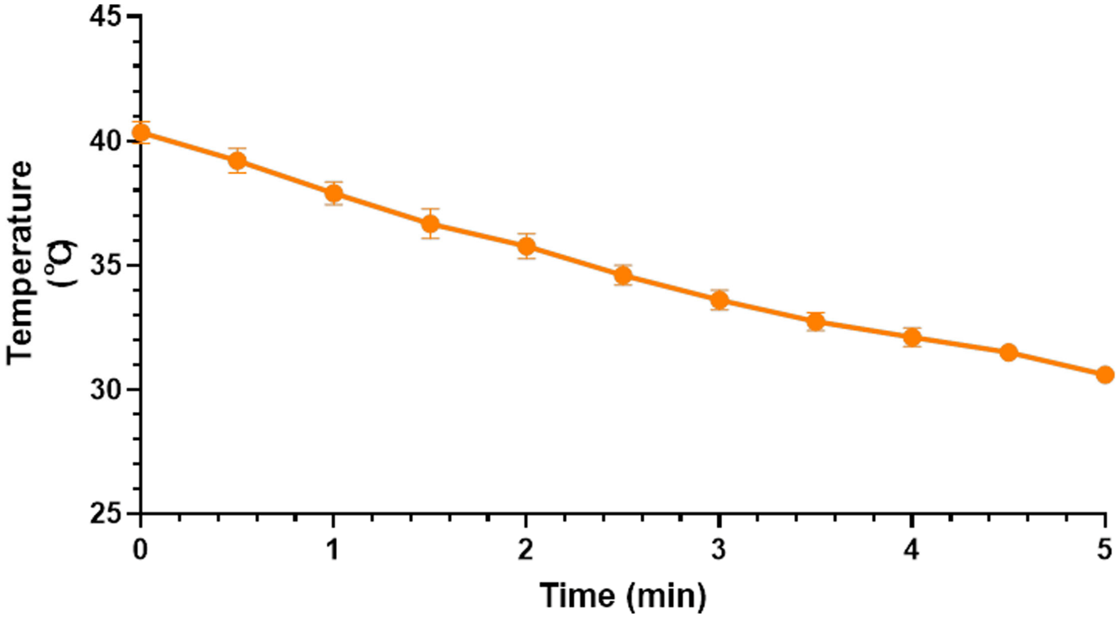

Figure S1. Temperature of the fish medium during the process of time-lapse imaging. Temperature of the medium was measured using a digital probe thermometer. Data are shown as mean ± standard deviation (n = 3).

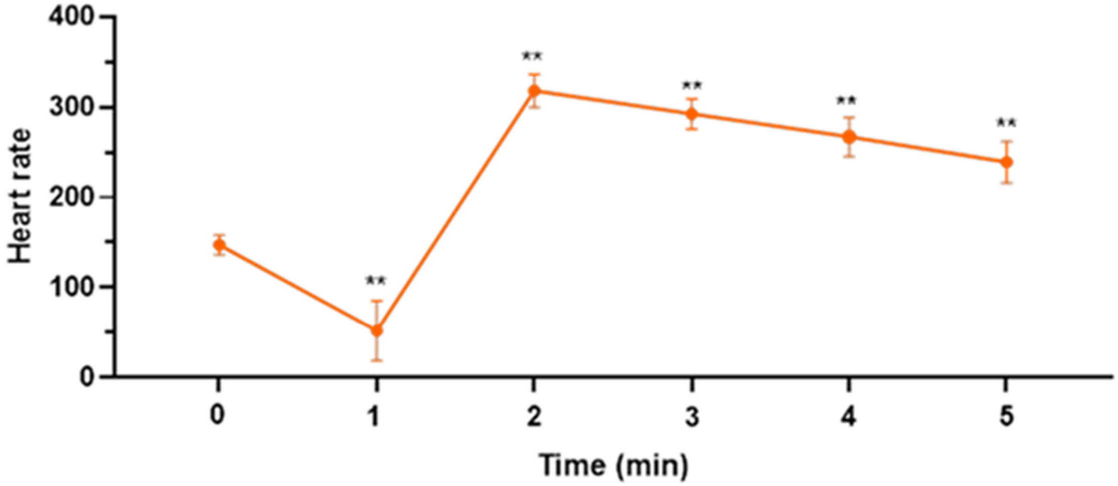

Figure S2. Heart rate of the zebrafish larvae exposed to heat stress decreased at first and then compensatively increased. Heart rates (beat/min) at the indicated time points were compared with that before exposure to heat stress (0 min). Data are shown as mean ± standard deviation (n = 3). **, $p < 0.01$.
